# Effect of Corrosive Media on the Chemical and Mechanical Resistance of IPS e.max^®^ CAD Based Li_2_Si_2_O_5_ Glass-Ceramics

**DOI:** 10.3390/ma15010365

**Published:** 2022-01-04

**Authors:** Anna Švančárková, Dagmar Galusková, Aleksandra Ewa Nowicka, Helena Pálková, Dušan Galusek

**Affiliations:** 1 FunGlass, Centre for Functional and Surface Functionalized Glass, Alexander Dubček University of Trenčín, Študentská 2, 911 50 Trencin, Slovakia; anna.savncarkova@tnuni.sk (A.Š.); dagmar.galuskova@tnuni.sk (D.G.); aleksandra.nowicka@tnuni.sk (A.E.N.); 2 Faculty of Chemical and Food Technology STU, Radlinského 9, 812 37 Bratislava, Slovakia; 3 Institute of Inorganic Chemistry, Slovak Academy of Sciences, Dúbravská cesta 9, 845 36 Bratislava, Slovakia; helena.palkova@savba.sk; 4 Joint Glass Centre of the IIC SAS, TnUAD and FChFT STU, 911 50 Trencin, Slovakia

**Keywords:** lithium disilicate, IPS e.max^®^ CAD, dental ceramics, corrosion, wear resistance

## Abstract

The influence of 4% acetic acid (pH~2.4) and an alkaline solution of NaOH (pH~10) on the corrosion resistance and micromechanical properties of disilicate crystals containing glass-ceramics (LS2-GC’s) is studied. Partially crystallized lithium metasilicate crystal containing glass-ceramics (LS-GC’s) are annealed to fully LS2-GC’s using a one stage and a two-stage heating to induce nucleation. Materials with various chemical and wear resistance are prepared. The content of the crystalline phase in the material annealed in the two-stage process A is 60.0% and increases to 72.2% for the material heated in the one-stage process B. The main elements leached in the acidic medium are lithium and phosphorus, while lithium, silicon, and phosphorus leached into the alkaline environment. Material B exhibits better chemical resistance to the corrosive influence of 4% acetic acid under quasi-dynamic conditions. In the alkaline corrosion medium, silicon is leached from material A faster compared to the material B. After prolonged exposure to acidic or basic environments, both materials show evidence of surface structural changes. A decrease of the sliding wear resistance is observed after corrosion in the acidic environment under dynamic conditions. In both materials, the wear rate increases after corrosion.

## 1. Introduction

Modern dental ceramics are characterized by their high durability in the oral cavity. The chemical durability of these biomaterials has to be higher than that of natural teeth to prevent the gradual development of corrosion-related surface defects, which could compromise their mechanical properties. 

LS2-GC’s are currently used for single and multi-unit dental restoration applications mainly intended for dental crowns, bridges, and veneers because they have a color similar to natural teeth and excellent mechanical properties. Numerous publications deal with the description of the mechanisms of nucleation and crystallization and the processes designed to achieve a fully dense, durable and translucent LS2-GC’s with a characteristic tooth color (see, e.g., [1,2,3]). 

The chemical durability of such glass-ceramic materials is good but may be influenced by many factors. These include not only the composition and microstructure of the material, but also the chemical nature of the corrosive medium, the exposure time, and the temperature. Prior to launching any synthetic dental material to the market, it is usually tested in a 4% acetic acid solution at 80 °C according to the ISO 6872 standard. The chemical durability is then evaluated mainly by weight loss, and only occasionally by the concentration of ions leached from the material into the corrosive medium. These tests were originally developed and applied to test the corrosion resistance of glass-ceramic heating plates and their conditions are irrelevant to the real oral environment. Any conclusions on the durability of dental materials based on experimental conditions that do not correspond to the conditions under which they are applied must be therefore taken with the utmost caution. Additionally, there are only few studies which relate the chemical durability of LS2-GC’s to their ion leaching characteristics [4,5]. 

The pH range experienced in the oral cavity ranges from 1 to 10 [6] with highly acidic conditions associated to the reflux of gastric fluids. More typically, the pH is related to the acidity of the beverages consumed and ranges from 3 to 8 for carbonated acidic sport and energy drinks, which contain stimulants such as guarana with a low pH [6]. On the other hand, other substances can have a very high pH, for example lima beans or soybeans (pH 12), spinach (pH 8.3) and antacids (pH 10–14) [7].

LS2-GC’s and feldspathic glass-ceramics are usually tested in an acetic acid solution at the temperature mentioned above. At pH < 5 the measured leaching rates decreased in the order Li^+^ > Si^4+^ > Ca^2+^ > Al^3+^ and were discussed in detail for the Dicor ceramics in Ref. [4]. A comparative study of different glass-ceramic materials was carried out by Jakovac: the highest leaching was measured in feldspathic glass-ceramics (IPS-Classic ceramic), while the LS2-GC’s (IPS-Empress) was the most corrosion resistant [5]. However, to the best of our knowledge, there are not many studies which tested LS2-GC’s under a variety of pH conditions and the temperatures typical for the oral cavity. The mechanism of glass-ceramic dissolution under dynamic conditions or in an alkaline environment are the subject of a small number of studies [4,7].

An important property of restorative materials is their hardness because it is a measure of the resistance to permanent surface indentation or penetration. Therefore, the science related to the corrosion of dental materials is focused on measuring hardness and tribological characteristics, as it describes the abrasiveness of a material to which the natural teeth may be submitted. 

There exists a large number of studies focused on lithium disilicate glass-ceramics. Most of them specifically target the influence of sample preparation conditions (thermal regime or initial composition) either on the mechanical properties [8,9] or chemical resistance of the final material [10]. Other studies focus only on the chemical resistance of the material tested under various conditions of the corrosion process [7]. Only a few studies have examined the properties of lithium disilicate glass ceramics from various angles. The study [11] looks at the properties of various lithium disilicate dental materials in a more complex way, comparing both the chemical resistance of the materials and their mechanical properties. 

This study aims to evaluating the corrosion resistance of IPS e.max^®^ CAD glass-ceramics, both in acidic and alkaline environments and at temperatures relevant to those in the oral cavity. Corrosion resistance was evaluated by a quantitative analysis of the elements leached from the test materials in corrosion solutions. The results are normalized with respect to the content of the elements in the material, yielding information on the mechanism of dissolution. This study also tries to determine which phase (crystal or glass) is primarily responsible for the durability of the material as a whole. The influence of corrosion on the sliding wear resistance of dental LS2-GC’s was monitored.

## 2. Materials and Methods

### 2.1. Sample Preparation

A commercial dental material, i.e., partially crystallized blocks of IPS e.max^®^CAD (Ivoclar Vivadent, Lichtenstein), was used for the study. The glass-ceramic blocks were cut into 12.4 × 7.5 × 1 mm plates and subjected to two different heat treatment processes. In process A, the dental material IPS e.max ^®^CAD was heated to 500 °C with a rate of 5 °C/min, held for 1 h and then ramped to 820 °C with a rate of 5 °C/min, with a 1 h isothermal dwell time. Afterwards, the samples were cooled at a rate of 5 °C/min to room temperature. In process B, the dental material was heated to 850 °C with a rate of 5 °C/min with a 1 h isothermal dwell time. Subsequently, the samples will be labelled according to the applied heat treatment processes (material A and material B). Prior to the corrosion tests, the samples were ground and polished (Buehler Ecomet300/Automet 300) to a 0.5 µm finish.

The composition of crystalline phases was determined by X-ray powder diffraction (XRD) using a PANalytical Empyrean DY1098 diffractometer (Panalytical, The Netherlands) using CuKα radiation (λ = 1.5405 Å). 5 wt.% of Si of analytical purity grade were added as an internal standard to quantify the amount of glass phase. The XRD patterns were recorded in the 2θ range from 10 to 40° with the scanning step size of 0.026°.

The chemical composition of the test materials was determined by inductively coupled plasma optical emission spectrometry (ICP OES Varian MPX, Varian Australia Pty Ltd., Mulgrave, Australia). To determine the chemical composition of the material, the samples were decomposed by microwave-assisted digestion (Microwave Speedway 4, Berghof). The ratio of acids used for microwave digestion were: 2 mL of HNO_3_ (67–69%, ANALPURE), 6 mL of HCl (30%, SUPRAPURE) and 0.5 mL of HF (47–51%, ANALPURE).

### 2.2. Corrosion Tests

The resistance of the prepared materials to corrosion in various environments was monitored both under static and *quasi*-dynamic conditions. For the tests, the samples were cleaned in deionized water in an ultrasonic bath and dried in an oven at 60 °C for 1 h. Then they were transferred to a plastic polypropylene (PP) bottle with 4 mL of the corrosive medium, fixing the surface/volume ratio to 0.33 cm^−1^. The *quasi*-dynamic tests were performed at 37 °C simulating the temperature in oral cavity [10,12] using two corrosive media: 4% acetic acid and a solution of 1 g/L of CAPS (3-cyclohexylaminopropanesulfonic acid) buffer with the pH value adjusted to 10 ± 0.05 by adding 1 mol/L of NaOH. The total duration of the test was 96 h and the corrosive medium was replaced every 12 h. Under static conditions, the samples were tested according to the ISO 6872 standard for the hydrolytic resistance of dental ceramic materials [13], i.e., the materials were exposed to 4% acetic acid at 80 °C for 16 h. The conditions of the corrosion tests are summarized in Table 1. After the test, the samples were rinsed with deionized water and dried in the oven at 60 °C for 1 h.

### 2.3. Analysis of Corrosion Solutions

The contents of the elements, namely Al, Li, Si, K and P, leached into the corrosive media were determined by ICP OES. Three parallel measurements were performed for each analysis and corrected to the mean blank values (*n* = 10), either in 4% acetic acid or a NaOH solution. The measured concentrations of each ion type in the corrosion solution, *c_i_* in mg/L, were then re-calculated to the cumulative amount of the leached element, *NL_i_*, mg/cm^2^, normalized to the leached surface area of the specimen and the mass fraction of an element in the sample according to Equation (1):

(1) $$NL_{i}^{t}=\frac{c_{i}}{w_{i}}\frac{V}{S}+NL_{i}^{t-\Delta t}$$

where *V* is the volume of corrosion medium in *L*, *w_i_* is the mass fraction of an element in the glass phase, *S* is the surface area of corroded material in cm^2^, and *t* is time in h.

### 2.4. Mechanical Properties 

The glass-ceramic samples were mechanically tested to determine the influence of corrosion on the sliding wear behavior. Two-body wear tests were conducted using a reciprocating horizontal tribometer. To better simulate real wear conditions of the dental material, these parameters were based on clinical experience and the literature [14]. Wear testing was carried out under dry conditions using the ball-on-flat technique, during which the abrasion ball is in contact with the surface of the test sample. The tribological partner was a highly polished alumina ball with a diameter of 6.35 mm, the applied load was 10 N, the sliding speed was 10 cm/s and the sliding distance was 50 m. The wear scar profiles were measured on a section of the sample with a Sensofar PLu Neox 3D optical profilometer (confocal microscope). After each wear test, the volume of material loss (mm^3^) was determined using this confocal microscope. For each sample, three parallel measurements were carried out and the average wear resistance was determined.

### 2.5. SEM Analysis 

The sample surfaces were examined using a Scanning Electron Microscope (SEM, JEOL JSM-7600 F). No polishing was applied after the corrosion tests, to preserve all corrosion induced features, such as pits or precipitated corrosion products. The samples were ultrasonically cleaned in acetone for 15 min, rinsed with deionized water and dried in an oven at 60 °C for 1 h. The surface was coated with carbon to prevent charging (JEOL JFC-1300 “AUTO Sputter Coater”). 

### 2.6. Attenuated Total Reflectance Infrared Spectroscopy 

Infrared spectra were measured on a Nicolet 6700 (Thermo Scientific) using the Smart Orbit™ horizontal single-reflection ATR accessory with a diamond, KBr beam splitter and DTGS detector. For each sample, 64 scans were recorded with a resolution of 4 cm^−1^. Spectra were collected using the Thermo Scientific OMNIC^TM^ software package. The spectra were normalized to the highest peak and an ATR correction was applied to the spectra.

## 3. Results

### 3.1. Characterization of the Prepared Materials

The chemical composition determined by ICP OES and the chemical composition declared by the manufacturer are compared in Table 2. As HF was used in sample dissolution for chemical analysis by ICP OES, yielding volatile SiF_4_, the content of silica could not be determined. The content of SiO_2_ was therefore calculated to sum up to 100% oxide content (marked with asterisk in Table 2).

The impact of the nucleation temperature on the materials’ properties was assessed by comparing the materials annealed with and without the nucleation step. The applied temperatures were chosen from the previously reported data [2], where the first temperature (500 °C) was applied to nucleate lithium metasilicate crystals, while the second temperature (820 °C or 850 °C) was used to grow the LS_2_ crystals. 

LS_2_ crystals form in the temperature interval 820 °C–850 °C [16] when the viscosity of residual glass is sufficient to the increase in the mobility of ions. This ion mobility allows the formation of LS_2_ crystals (Equation (2)), with a composition close to the chemical composition of the glassy matrix [1].

Li_2_SiO_3_ (crystal) + SiO_2_ (glass) → Li_2_Si_2_O_5_ (crystal) (2)

With the additional nucleation step, we focused on obtaining a higher fraction of crystalline phase. Only the influence of temperature on the process of crystallization was monitored and not the duration of the crystallization. 

Yuan et al. [17] have shown that the annealing time had no significant effect on the phase composition of crystallized LS_2_-GC’s. However, they confirmed a significant effect of the annealing time on the size of LS_2_ crystals.

**Figure 1:**
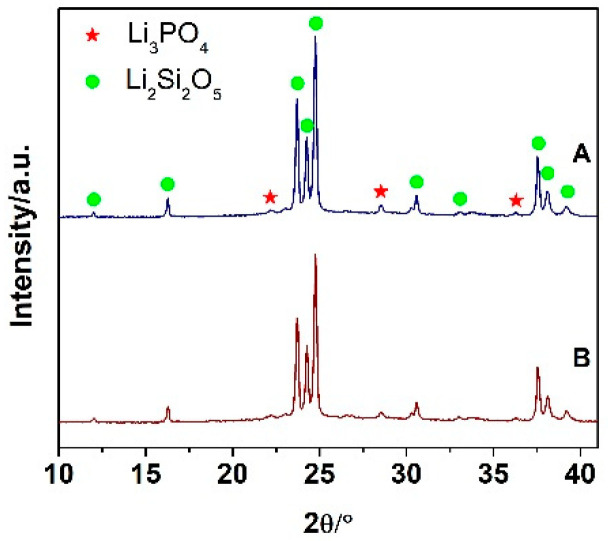
XRD patterns acquired from the materials A and B.

The XRD patterns of the specimens A and B are shown in Figure 1. Their major XRD peaks are attributed to lithium disilicate (Li_2_Si_2_O_5_, LS_2_, ICDD 40-0376); the minor phase corresponds to lithium phosphate (Li_3_PO_4_, LP, ICDD 45-0747). Despite the different heat treatments, the phase composition seems to be the same, confirming that the inclusion of the nucleation step and different crystallization temperature had no significant effect on the phase composition of the prepared LS_2_-GC’s measured by XRD.

However, subsequent Rietveld refinement with the internal standard (5.0 wt.% of Si) aimed at determining the content of glassy phase confirmed a higher content of crystalline phases in the material B annealed in the one-stage process at 850 °C for 1 h (Table 3). This material contained 65.5 wt.% of LS_2_ and 6.3% of LP. Glass phase accounted for 27.8 wt.% of the material. The material A, heated in a two-stage regime, contained 40.0 wt.% of the glass (Table 3). The material also contained 55.0 wt.% of LS_2_ and 5.0wt% of LP indicating that the higher annealing temperature led to a higher overall content of analyzed crystalline phases.

**Figure 2:**
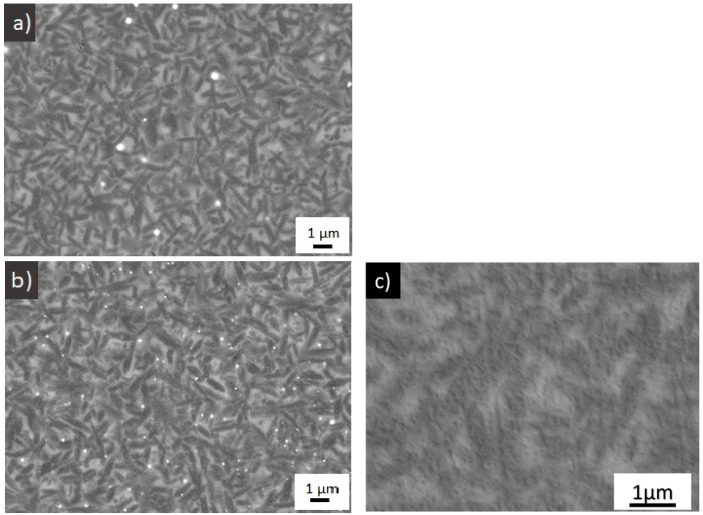
SEM micrographs of (**a**) material A (**b**) material B and (**c**) material B—higher magnification.

Figure 2 shows SEM micrographs of the microstructures of both materials after the heat treatment. The content and shape of crystals appears to be approximately the same. Based on the contents of crystalline phases estimated from the Rietveld refinement, we calculated the chemical composition of glass phase in both annealed materials (Table 4).

The corrosion of glass-ceramics is complex because both glassy and crystalline phases of various compositions are present. They dissolve at different rates and with possibly different mechanisms. Dissolution of the glass matrix can also lead to the release of less soluble crystals into the solution, further affecting the ion release kinetics [18,19]. After exposure to both acidic and alkaline media, discreet pits in the residual glass were observed, indicating its corrosion. Following the SEM analysis of corroded surfaces, we assumed that the glass primarily dissolved during the corrosion process, and all elements detected in the corrosion media were leached exclusively from the glass. The composition of the glass phase was then used to calculate the *NL*_i_ values.

### 3.2. Corrosion Process in an Acidic Environment

**Figure 3:**
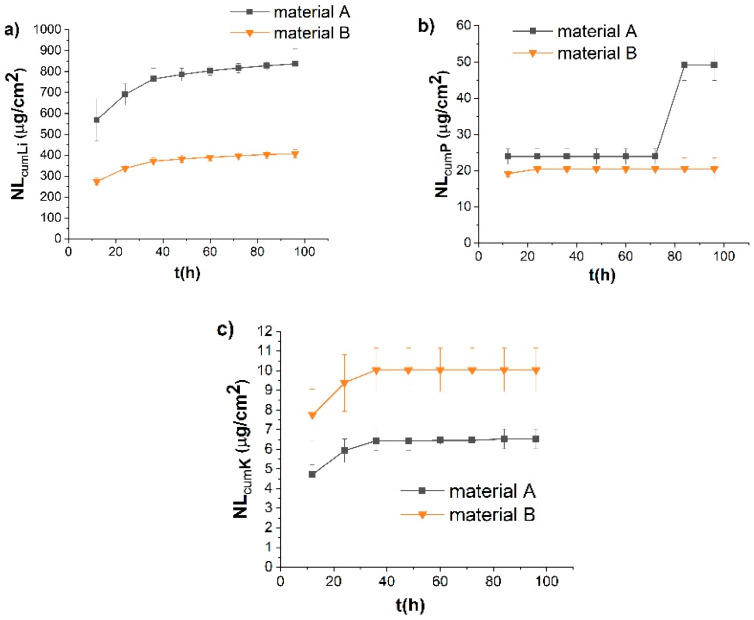
Time dependence of the ions released from the materials corroded in 4% acetic acid during the quasi-dynamic test, expressed in cumulative NL values of (**a**) Li, (**b**) P and (**c**) K.

In an acidic environment, the predominant reaction is an ion exchange between the protons in the solution and network modifiers in the sample. Li and P were detected as major elements leached by the acidic corrosion solution during the *quasi*-dynamic tests (Figure 3). This trend was observed in both tested materials. Anusavice [20] showed that Li^+^ is selectively leached from the glass-ceramic surface through an ion exchange with H^+^ or H_3_O^+^ from the solution during the corrosion of lithium-containing glass-ceramics in an acidic aqueous environment. In our case, and for both materials, Li elements were leached in the acidic solution at the highest rates in comparison with other leached ions. For the material annealed in the two-stage process, the amount of Li after 24 h was 690 ± 51 µg/cm^2^ (cumulative NL value based on the amount of Li in the glassy phase in the material); after the 96 h corrosion test, it reached 837 ± 70 µg/cm^2^. Only 337 ± 16 µg/cm^2^ and 408 ± 20 µg/cm^2^ of Li was leached from the material B after 24 h and 96 h, respectively. Material A contains considerably more Li (6.6 wt%) in the glass phase compared to material B (1.5 wt%), which in turn leads to a faster release of Li from the glass phase. Li is known as a network modifier in glass [21]. Its higher content in the glass phase of material A disrupts the crosslinking of the glass structure to a greater extent. Consequently, this leads to a higher solubility of the material A. The lower connectivity of the silicate network is also reflected in the increased release of silicon from the material A compared to material B in an alkaline environment. The content of P is considerably higher in the glass phase of the material B (15.9 wt% compared to 3.1 wt% in the material A), which led to a higher amount of P released from the material B into the acidic corrosion solution (0.47 mg/L of P leached from glass-ceramic B after 24 h, while only 0.11 mg/L were leached from the material A). However, when re-calculated to the NL values, the release rates were approximately the same after the first 72 h of testing (23.9 ± 2.2 µg/cm^2^ for material A, vs. 20.5 ± 0.6 µg/cm^2^ for material B). Due to the corrosion, the release rate of P from the material A even increased to 49 ± 4 µg/cm^2^, while the amount of released P from material B remained unchanged. The reason is most likely the lower hydrolytic resistance of glassy phase in material A, which can leach both P and Li ions faster. This delayed acceleration of the release of P from material A can be due to the longer-term action of the acid on the material, which leads to a more extensive disruption of the glass network compared to material B and thus a faster release of the P. 

The cumulative amount of K in the solution of 4% acetic acid was 6.5 ± 0.5 µg/cm^2^ after the 96 h dynamic test for material A; and 10.0 ± 1.1 µg/cm^2^ for material B. The rates of K release for both materials are comparable, but significantly lower than the released amounts of Li or P. The concentration of Al and Si leached in the acetic acid during the *quasi*-dynamic test was below the limit of detection for both tested materials. A higher resistance to corrosion in an acidic environment was measured for material B annealed in the one-stage process with a higher crystallization temperature and with a lower content of the residual glass phase. This suggests that the phase which dissolved in the 4% acetic acid was glass. 

**Figure 4:**
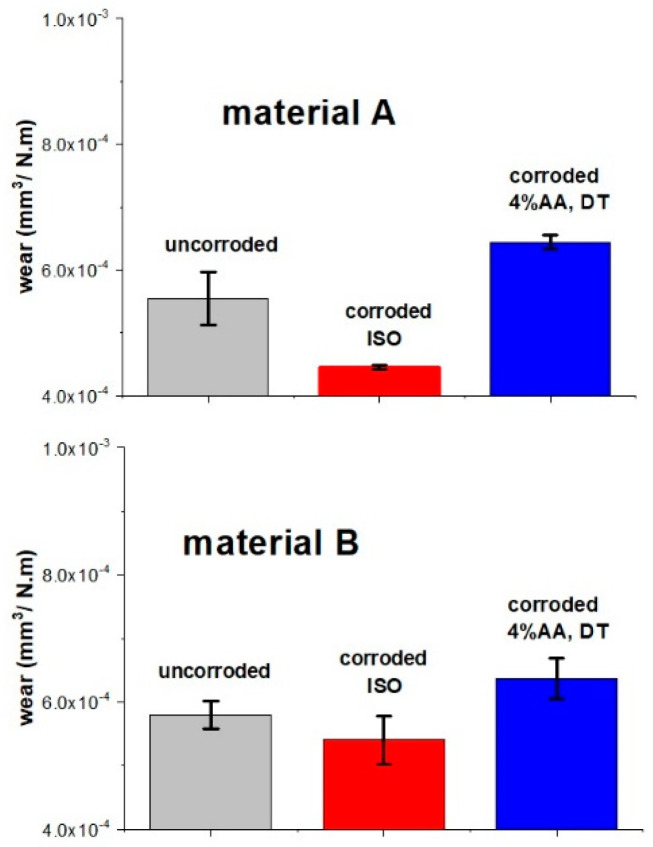
Wear rates of LS_2_-GCs in a uncorroded sample, after static corrosion for 16 h at 80 °C (ISO), and a dynamic test for 96 h (4% AA, DT) in 4% acetic acid for material A and material B.

**Figure 5:**
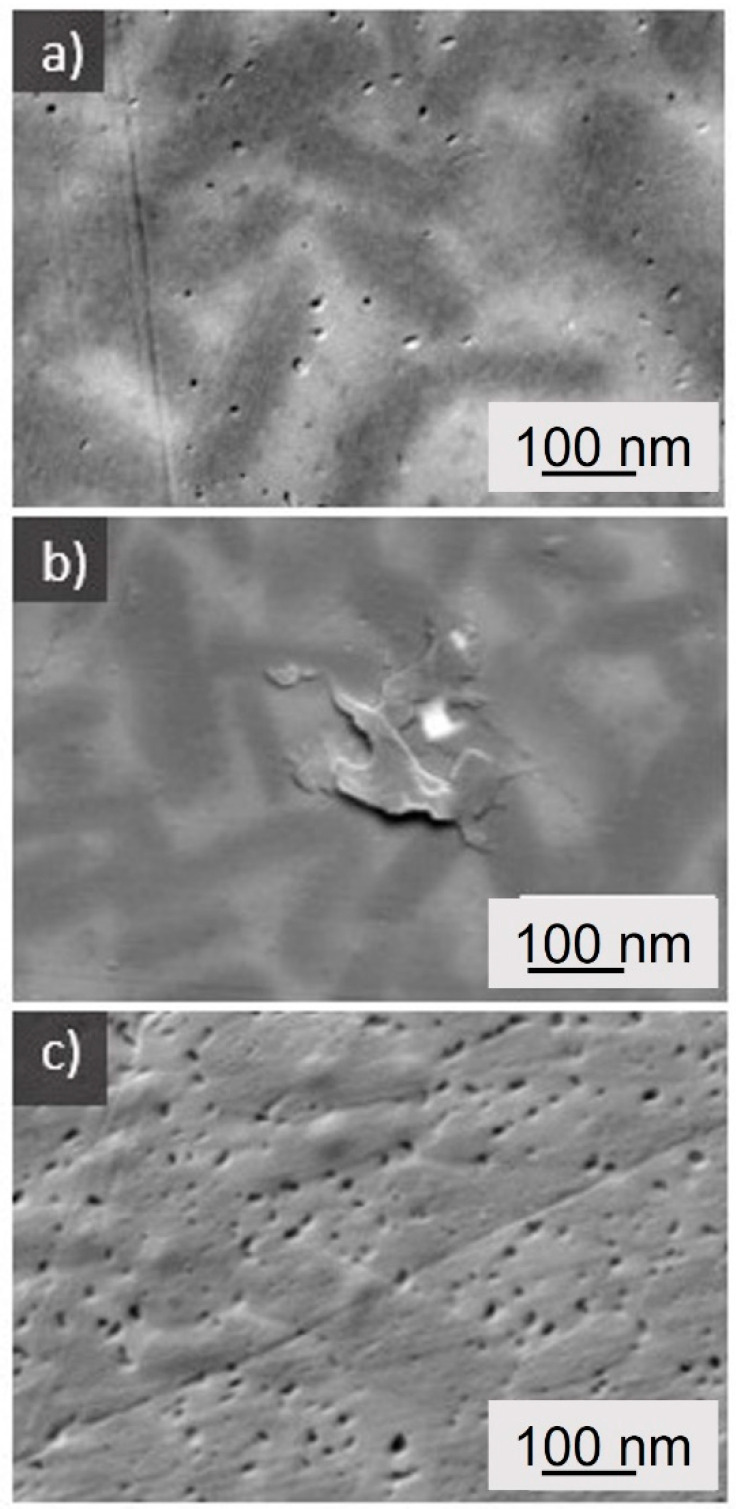
Representative SEM images for material A: (**a**) after the dynamic test for 96 h in 4% acetic acid (**b**) after the static test for 16 h at 80 °C (ISO 6872) (**c**) after the dynamic test in an alkaline solution (pH 10).

The influence of the acidic medium or annealing process on dental material was further evaluated by monitoring the change of mechanical properties before and after corrosion, especially the resistance against sliding wear. After annealing, both samples exhibited very similar wear rates (5.5 ± 0.4×10^−4^ mm^3^/Nm for A and 5.8 ± 0.2×10^−4^ mm^3^/Nm for B, see Figure 4). After dynamic corrosion in a 4% acetic acid, the wear rates for both materials increased to an identical wear rate of 6.4×10^−4^ mm^3^/Nm. Such a decrease of corrosion resistance was attributed to the partial dissolution of the glass phase during corrosion. Pores formed on the surface as the result of dissolution of the glassy phase and confirmed by SEM analysis (Figure 5), decreased the resistance of the material’s surface to penetration of the alumina ball used as a counter body during the wear resistance test. Such a decrease can eventually lead to gradual accumulation of damage of LS_2_-GCs dental materials.

Interestingly, the corrosion test performed in accordance with the ISO standard in both cases led to an increase in wear resistance: a 6.9% decrease in the wear rate was measured for material B, and an 18.2% decrease in the wear of material A.

The SEM analysis (Figure 5) confirmed that the surface of the dental material contained numerous micropores after dynamic corrosion in the acetic acid (Figure 5a). A coating of unknown composition was observed on the surface of the ceramics after the static corrosion test (16 h) in the acidic medium (Figure 5b). This coating, presumably acting as a lubrication layer, was considered as the possible cause of the decreasing wear rates after this static corrosion tests. 

A much more pronounced increase of the surface porosity was observed after corrosion in the alkaline solution (Figure 5c) compared with the material corroded in the acidic environment. As indicated by the results of the chemical analysis of the corrosion solution, the pores were formed as the results of dissolution of the glass network by an OH^−^ attack on the Si–O bonds. This effect was observed in both samples.

**Figure 6:**
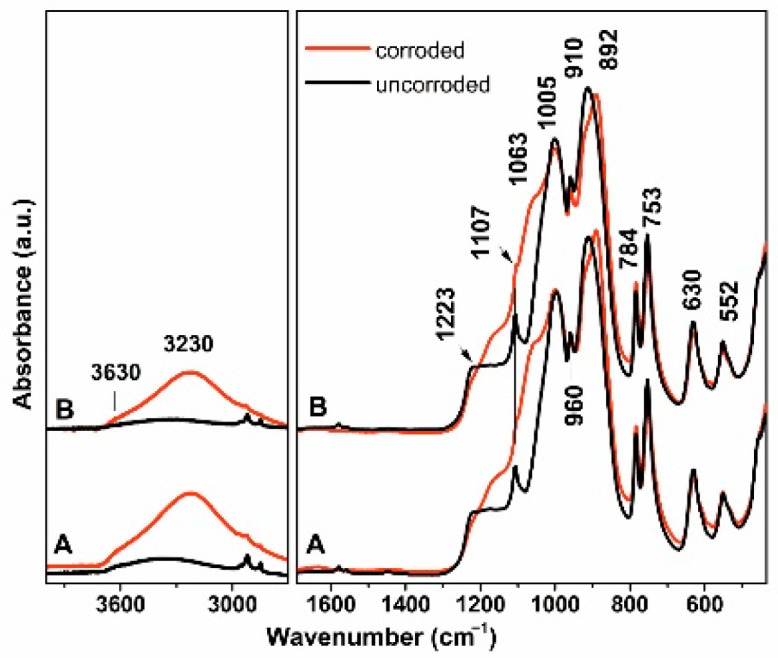
ATR-FTIR spectra of the studied samples prior and after corrosion process.

The results obtained from infrared spectra measurements were used to evaluate changes in the composition of material A and material B after subjecting the samples to the acid leaching process (Table 5, Figure 6). IR spectra of both samples prior to treatment exhibited almost identical spectral features. A broad low intensity band near 3400 cm^−1^ with an indistinct shoulder near 3630 cm^−1^ was observed in the high wavenumber region. The band belongs to stretching vibrations of O–H groups (νO–H) from aluminol or silanol groups variously bonded in the sample. Chemical analysis revealed between 11.7 and 13.5 wt.% of aluminum oxide in the samples (Table 4). However, no crystalline Al-containing phase was detected by X-ray powder diffraction, or these phases were below the detection threshold (Table 3), indicating the possible incorporation of Al in the amorphous (glassy) phase with terminal Al–OH groups at the specimen surface. 

In the spectral region below 2000 cm^−1^, several bands of various intensities were observed in the spectra. Crystalline LS_2_ as the dominating siliceous phase was determined by XRD (Table 3): consequently, the observed bands could be attributed to the various vibrations of this phase. Based on previous works discussing IR spectra of LS_2_-GC’s [22,23], the sharp components at 1107, 1005, 960 and 910 cm^−1^ belong to various modes of asymmetric Si–O stretching vibrations (ν_as_Si–O), the bands near 784, 753 and 629 cm^−1^ to symmetric Si–O vibrations (ν_s_Si–O), while bending Si–O vibrations (δSi–O) can be found at lower wavenumbers below 600 cm^−1^. Although amorphous phases considerably contribute to the composition of both tested materials (Table 3), the absorption bands originating from the glass were most likely overlapped with more intensive bands of crystalline LS_2_. A similar assumption could be adopted for crystalline LP with the content too low to be identified within absorption bands of the dominating phase. The most intensive bands of LP occur in the spectral region 1200–1050 cm^−1^ and they belong to the asymmetric stretching vibration from (PO_4_)^3−^ ionic group [24,25].

Treatment in the acidic environment evoked a qualitative change in the shape of the infrared spectra. The relative intensity of the broad band between 3600–3100 cm^−1^ increased and the band was shifted to lower wavenumbers, indicating an increasing contribution of O–H groups in the samples. A significant broadening of the bands above 1005 cm^−1^ could indicate an increasing contribution of the amorphous phase formed in the corroded samples [26,27,28]. At these wavenumbers, the absorption bands of three dimensional SiO_2_ were detected. A shift of the sharp band near 910 cm^−1^ to 892 cm^−1^ could be attributed to changes in the sample structure. The rest of the spectra did not display any significant changes or the formation of new bands.

### 3.3. Corrosion Process in Alkaline Environment

**Figure 7:**
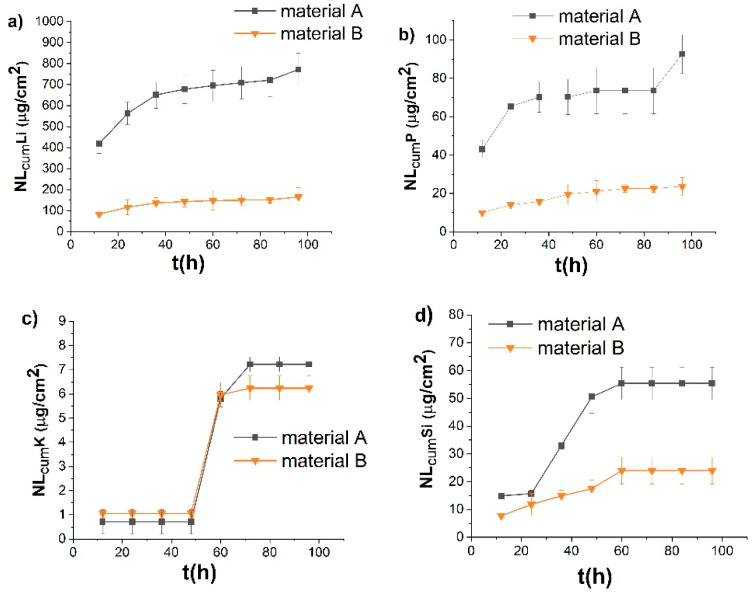
Time dependence of the normalized leaching of ions released from the materials studied in NaOH solution during the quasi-dynamic analysis, expressed in cumulative NL values of (**a**) Li, (**b**) P, (**c**) K and (**d**) Si.

In the alkaline environment, the hydroxyl ions in the solution attack the glass structure resulting in its general dissolution. In their studies, McCracken et al. [29] and Charles [30] reported general rules where acid exposure caused only a small surface damage, while alkaline solution caused extensive surface changes in the amorphous phase of glass-ceramics. This effect in an alkali corrosion solution (for a pH 9 or higher) is caused by the dissolution of the glass network by an OH^−^ attack on the Si–O bonds. We observed Si elements leached in the alkali corrosion solution as the result of cleavage of Si–O bonds in the glass phase (Figure 7). This process was indicated by SEM analysis (Figure 5c), where an extensive damage of the glassy phase in glass ceramics was observed. In the alkaline environment, Li, Si and P ions were leached extensively. The amount of leached Al was below the detection limit. Si was leached from material A at a higher rate (16 ± 1 µg/cm^2^ after 24 h, in comparison to 12 ± 4 µg/cm^2^ for material B), as the result of a higher content of glass in the material heated in the two-stage process at a lower maximum temperature. After the complete corrosion test in the alkaline solution, 55 ± 6 µg/cm^2^ of Si leached from material A and 24 ± 5 µg/cm^2^ from material B. The amount of Li leached from material A was 563 ± 54 µg/cm^2^ after 24 h and 771 ± 79 µg/cm^2^ after 96 h of corrosion. Material B leached 117 ± 35 µg/cm^2^ of Li after 24 h, and 165 ± 45 µg/cm^2^ at the end of the test. The overall higher content of Li in the glass phase of material A was reflected in the faster release of Li into the alkaline environment from this material. 

At the end of testing in the alkaline solution, the cumulative NL value for P was 93 ± 10 µg/cm^2^ (material A) and 24 ± 5 µg/cm^2^ (material B). Despite the significantly higher proportion of P in the glass phase of material B, we observed a faster release of this element from material A. This may be due to the faster degradation of the glass phase in material with a higher glass content. As the Si–O–Si networks dissolve more rapidly in material A, this leads to a faster release of the elements built into this silicate network. It is visible especially in the last stage of corrosion, after 96 h, when we see a sudden jump in the dissolution rate of P and Li from the material A. 

A very low amount of P in the solutions after corrosion was measured both for material A (7.2 ± 0.3 µg/cm^2^), and B (6.2 ± 0.5 µg/cm^2^). 

**Figure 8:**
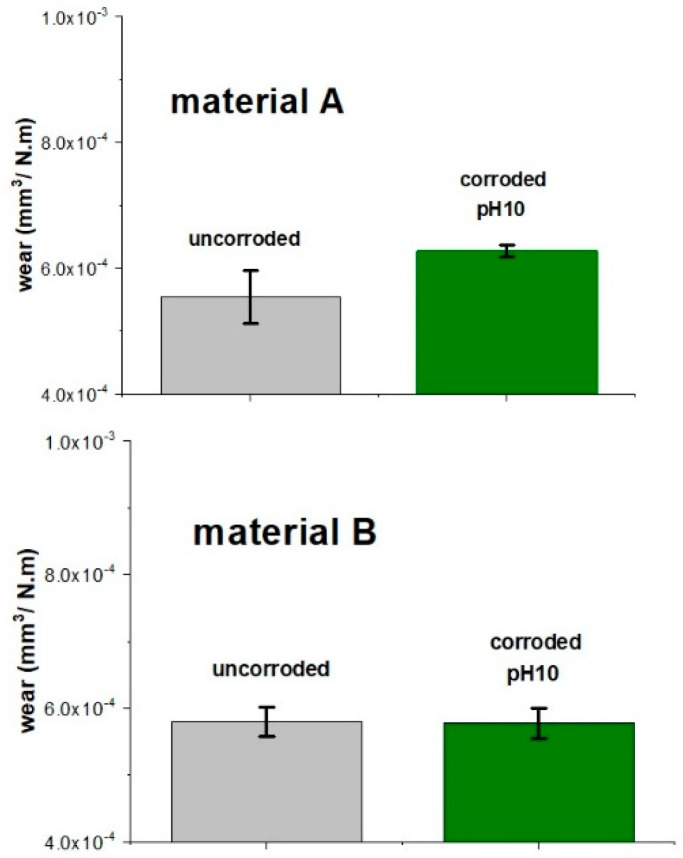
Wear rates of LS_2_-GCs measured in the uncorroded samples and in samples corroded under dynamic conditions in NaOH solution for material A and material B.

The wear rate of the material sintered in the two-stage process increased after 96 h of *quasi*-dynamic testing in an alkaline solution of NaOH from 5.5×10^−4^ mm^3^/Nm for uncorroded samples to 6.3 ×10^−4^ mm^3^/Nm for corroded material (Figure 8). The dynamic test in the NaOH solution had no influence on the wear resistance of material B, sintered in a one-stage process. However, corrosion in an alkaline environment led to a decrease in the wear resistance of the material heated at a lower temperature, i.e., the material with a higher glass phase content, due to faster dissolution of the glass phase. 

### 3.4. Toxicity and Chemical Solubility of the Materials

From the toxic viewpoint, the daily allowable intake of lithium is a total amount of 2 mg [31]. Assuming that 28 teeth received full crowns made from our test material, with a total surface area of 74 cm^2^, the maximum daily amount (after 24 h) of Li released from material A in 4% acetic acid at 37 °C was 777.0 µg and 291.8 µg in the alkaline solution. For material B, sintered in a one-stage process, it was 144.6 µg during the same conditions in an acidic environment, and 277.8 µg in a NaOH solution. We can therefore conclude that the amount of Li leached from both sintered materials, in both an acidic and alkaline environment, was far below the acceptable daily doses acquired through food-intake.

According to Anusavice [20], ceramics are considered to be the most durable of all dental materials. The scientific documentation of IPS e.max^®^ CAD declares a chemical solubility of 40 ± 10 µg/cm^2^ (during an ISO test). This chemical solubility is far below the threshold value specified by the ISO 6872 test (˂100 µg/cm^2^). We observed the same chemical solubility for both materials, approx. 21.6 µg/cm^2^, after ISO corrosion. 

Any concerns related to possible health risks associated with corrosion and leaching of constituents of LS_2_-GC’s can be considered to be irrelevant except for allergic reactions to the material. However, a corrosion over an extended period of time both in acidic and basic environment can cause extensive damage, aggravated by the fact that unlike natural teeth, these materials are not subject of any self-healing mechanisms. Such damage may result in excessive wear and gradual formation of surface defects. 

## 4. Conclusions

1. Different heat treatments of IPS e.max^®^ CAD resulted in materials with different ratios of crystalline and amorphous phases. The higher crystallization temperature led to higher crystalline content.
2. The pH of the corrosion solution affected the ion leaching process significantly. Li and P were primarily leached in the acidic environment. In the alkaline NaOH solution with the pH = 10, primarily Li, Si and P elements were leached from the materials.
3. After *quasi*-dynamic corrosion for 96 h at 37 °C in 4% acetic acid, the highest ion elution was shown in the material heat treated at 820 °C. The material heat-treated at the maximum temperature of 850 °C was more resistant to *quasi*-dynamic corrosion in an acidic medium.
4. This study confirmed that exposure to an alkaline environment results in a breakdown of the silica network as compared with exposure to an acidic solution, which resulted in selective ionic leaching of the amorphous phase (mostly Li elements).
5. The results of this in vitro studies on chemical corrosion suggest that acidic environments adversely affect the wear resistance of tested dental materials. Corrosion in an acidic environment weakens LS_2_-GCs, IPS e.max^®^ CAD.
6. The Li elution was not toxicologically significant.

## Figures and Tables

**Table 1:** Corrosion test conditions.

Solution	pH (21.3 °C)	Conditions	Temperature	Time Interval (Test Duration)
4% acetic acid	2.4	*quasi*-dynamic	37 °C	12 h (96 h)
4% acetic acid *	2.4	static	80 °C	16 h
NaOH solution	10	*quasi*-dynamic	37 °C	12 h (96 h)

* ISO 6872 standard.

**Table 2:** Chemical composition of IPS e.max^®^ CAD measured by ICP OES and according to the literature.

Oxides	wt.% of Oxide (ICP OES)	wt.% of Oxide Ref. [15]
Li_2_O	14.3 ± 0.8	11.0–19.0
P_2_O_5_	4.3 ± 0.2	0.0–11.0
Al_2_O_3_	4.8 ± 0.1	0.0–5.0
K_2_O	9.1 ± 1.3	0.0–13.0
SiO_2_	67.5 *	57.0–80.0

* summed up to 100%.

**Table 3:** Phase composition of material A and B determined by the Rietveld analysis.

wt% of Phases	Material A	Material B
Li_2_Si_2_O_5_	55.0	65.5
Li_3_PO_4_	5.0	6.3
amorphous phase	40.0	27.8

**Table 4:** Estimated chemical composition of glass phase in tested materials.

Oxides	wt.% of Oxide Sample A	wt.% of Oxide Sample B
Li_2_O	6.6	1.5
P_2_O_5_	3.1	15.9
Al_2_O_3_	11.7	13.5
K_2_O	22.3	25.7
SiO_2_	56.3	43.4

**Table 5:** Positions and assignment of bands in ATR spectra.

Wavenumber (cm^−1^)	Bands Assignement
Before corrosion	
3400 with an indistinct shoulder near 3630	stretching vibrations of O-H groups
1107, 1005, 960, 910	asymmetric stretching vibrations of Si–O bond
784, 753 and 629	symmetric Si–O vibrations
below 600	bending Si–O vibrations
1200–1050	asymmetric stretching vibration (PO_4_)^3−^
After corrosion according ISO 6872 test	
3600-3100	increasing contribution of O–H groups
>1005	increasing contribution of the amorphous phase
band shift 910 to 892	changes in the sample structure

## Data Availability

The data presented in this study are available on request from the corresponding author.

## References

[1] Höland W., Beall H.G. (2012). Principles of designing glass-ceramic formation. Glass-Ceramic Technology.

[2] Huang S., Huang Z., Gao W., Cao P. (2015). Trace phase formation, crystalization kinetics and crystallographic evolution of a lithium disilicate glass probed by synchrotron XRD technique. Sci. Rep..

[3] Rampf M., Dittmer M., Ritzberger C., Schweiger M., Höland W. (2015). Properties and crystallization phenomena in Li_2_Si_2_O_5_–Ca_5_(PO_4_)3F and Li_2_Si_2_O_5_–Sr_5_(PO_4_)3F glass–ceramics via twofold internal crystallization. Front. Bioeng. Biotechnol..

[4] Anusavice K.J., Zhang N.Z. (1997). Chemical durability of Dicor anf lithia-based glass-ceramics. Dent. Mater..

[5] Jakovac M., Živko-Babić J., Ćurković L., Aurer A. (2006). Measurement of ion elution from dental ceramics. J. Eur. Ceram. Soc..

[6] Swain M.V. (2014). Impact of oral fluids on dental ceramics: What is the clinical relevance?. Dent. Mater..

[7] Esquivel-Upshaw J.F., Dieng F.Y., Clark A.E., Neal D., Anusavice K.J. (2013). Surface degradation of dental ceramics as a function of environmental pH. J. Dent. Res..

[8] Li D., Guo J.W., Wang X.S., Zhang S.F., He L. (2016). Effect of crystal size on the mechanical properties of a lithium disilicate glass-ceramic. Mater. Sci. Eng. A.

[9] Quinn J.B., Sundar V., Lloyd I.K. (2003). Influence of microstructure and chemistry on the fracture tougness of dental ceramics. Dent. Mater..

[10] Milleding P., Haraldsson C., Karlsson S. (2002). Ion leaching from dental ceramics during static in vitro corrosion testing. J. Biomed. Mater. Res..

[11] Ohashi K., Kameyama Y., Wada Y., Midono T., Miyake K., Kunzelmann K., Nihei T. (2017). Evaluation and comparison of the characteristics of three pressable lithium disilicate glass ceramic materilas. Int. J. Dev. Res..

[12] Kukiattrakoon B., Hengtrakool C., Kedjarune Leggat U. (2011). Effect of Acidic Agents on Surface Rougness of Dental Ceramics. Dent. Res. J..

[13] (1995). International Standards for Dental Ceramics.

[14] Zheng J., Zhou Z.R. (2007). Friction and wear behavior of human teeth under various wear conditions. Tribol. Int..

[15] Ivoclar Vivadent Scientific Documentation IPS e.max CAD. https://www.ivoclar.com/en_li/products/digital-processes/ips-e.max-cad.

[16] Holand W., Apel E., van’t Hoen C., Rheinberger V. (2006). Studies of cystal phase formations in high-strenght lithium disilicate glass-ceramics. J. Non-Cryst. Solids.

[17] Yuan K., Wang F., Gao J., Sun X., Deng Z., Wang H., Chen J. (2013). Effect of sintering time on the microstructure, flexural strenght and translucency of lithium disilicate galss-ceramics. J. Non-Cryst. Solids.

[18] Garai M., Karmakar B. (2020). Zr^4+^-controlled nucleation and microstructure in Si-Mg-Al-K-B-F glass ceramic sealant (solid oxide fuel cell). Mater. Today Energy.

[19] Garai M., Reka A.A., Karmakar B., Molla A.R. (2021). Microstructure–mechanical properties of Ag0 /Au0 doped K–Mg–Al–Si–O–F glass-ceramics. R. Soc. Chem. Adv..

[20] Anusavice K.J. (1992). Degradability of dental ceramics. Adv. Dent. Res..

[21] Macon A.L.B., Jacquemin M., Page S.J., Li S., Bertazzo S., Stevens M.M., Hanna J.V., Jones J.R. (2016). Lithium-silicate sol–gel bioactive glass and the effect of lithium precursor on structure–property relationships. J. Sol-Gel Sci. Technol..

[22] Fuss T., Moguš-Milanković A., Ray C.S., Lesher C.E., Youngman R., Day D.E. (2006). Ex situ XRD, TEM, IR, Raman and NMR spectroscopy of crystallization of lithium disilicate glass at high pressure. J. Non-Cryst. Solids.

[23] Ye J., Wen C., Wu J., Wen N., Sa B., Zhang T. (2019). Mechanical and bioactive properties of lithium disilicate glass-ceramic mixtures synthesized by two different methods. J. Non-Cryst. Solids.

[24] Salah A.A., Jozwiak P., Zaghib K., Garbarczyk J.E., Gendron F., Mauger A., Julien C. (2006). FTIR fearures of lithium-iron phosphates as electrode materials for rechargeable lithium batteries. Spectrochim. Acta Parta A.

[25] Moustafa M.G., Sanad M.M.S., Hassaan M.Y. (2020). NASICON-type lithium iron germanium phosphate glass ceramic nanocomposites as anode materials for lithium ion batteries. J. Alloys Compd..

[26] Sasmal N., Garai M., Karmakar B. (2015). Preparation and characterization of novel foamed porous glass-ceramics. Mater. Charact..

[27] Garai M., Sasmal S., Molla A.R., Singh S.P., Tarafder A., Karmakar B. (2014). Effects of nucleating agents on crystallization and microstructure of fluorophlogopite mica-containing glass–ceramics. J. Mater. Sci..

[28] Garai M., Sasmal N., Molla A.R., Karmakar B. (2015). Structural effects of Zn^+2^/Mg^+2^ ratios on crystallization characteristics and microstructure of fluorophlogopite mica-containing glass-ceramics. Solid State Sci..

[29] McCracken W.J., Clark D.E., Hench L.L. (1982). Aqueous durability of lithium disilicate glass-ceramics. American Ceram. Soc. Bull..

[30] Charles R.J. (1958). Static fatigue of glass. J. Appl. Phys..

[31] Goyer R.A., Amdur M.O., Doull J., Klaassen C.D. (1984). Toxic effects of metals: Lithium. Toxicology-The Basic Science of Poisons.

